# Biodiversity of the microbial mat of the Garga hot spring

**DOI:** 10.1186/s12862-017-1106-9

**Published:** 2017-12-28

**Authors:** Alexey Sergeevich Rozanov, Alla Victorovna Bryanskaya, Timofey Vladimirovich Ivanisenko, Tatyana Konstantinovna Malup, Sergey Evgenievich Peltek

**Affiliations:** 1 Federal Research Center Institute of Cytology and Genetics, the Siberian Branch of the Russian Academy of Sciences, Novosibirsk, Russia; 2 Novosibirsk State University, Novosibirsk, Russia

**Keywords:** Garga, Metagenome, Bacterial mats, Microbial community, Hot springs

## Abstract

**Background:**

Microbial mats are a good model system for ecological and evolutionary analysis of microbial communities. There are more than 20 alkaline hot springs on the banks of the Barguzin river inflows. Water temperature reaches 75 °C and pH is usually 8.0–9.0. The formation of microbial mats is observed in all hot springs. Microbial communities of hot springs of the Baikal rift zone are poorly studied. Garga is the biggest hot spring in this area.

**Results:**

In this study, we investigated bacterial and archaeal diversity of the Garga hot spring (Baikal rift zone, Russia) using 16S rRNA metagenomic sequencing. We studied two types of microbial communities: (i) small white biofilms on rocks in the points with the highest temperature (75 °C) and (ii) continuous thick phototrophic microbial mats observed at temperatures below 70 °C. Archaea (mainly Crenarchaeota; 19.8% of the total sequences) were detected only in the small biofilms. The high abundance of Archaea in the sample from hot springs of the Baikal rift zone supplemented our knowledge of the distribution of Archaea. Most archaeal sequences had low similarity to known Archaea. In the microbial mats, primary products were formed by cyanobacteria of the genus *Leptolyngbya*. Heterotrophic microorganisms were mostly represented by Actinobacteria and Proteobacteria in all studied samples of the microbial mats. Planctomycetes, Chloroflexi, and Chlorobi were abundant in the middle layer of the microbial mats, while heterotrophic microorganisms represented mostly by Firmicutes (Clostridia, strict anaerobes) dominated in the bottom part. Besides prokaryotes, we detect some species of Algae with help of detection their chloroplasts 16 s rRNA.

**Conclusions:**

High abundance of Archaea in samples from hot springs of the Baikal rift zone supplemented our knowledge of the distribution of Archaea. Most archaeal sequences had low similarity to known Archaea. Metagenomic analysis of microbial communities of the microbial mat of Garga hot spring showed that the three studied points sampled at 70 °C, 55 °C, and 45 °C had similar species composition. Cyanobacteria of the genus Leptolyngbya dominated in the upper layer of the microbial mat. Chloroflexi and Chlorobi were less abundant and were mostly observed in the middle part of the microbial mat. We detected domains of heterotrophic organisms in high abundance (Proteobacteria, Firmicutes, Verrucomicrobia, Planctomicetes, Bacteroidetes, Actinobacteria, Thermi), according to metabolic properties of known relatives, which can form complete cycles of carbon, sulphur, and nitrogen in the microbial mat. The studied microbial mats evolved in early stages of biosphere formation. They can live autonomously, providing full cycles of substances and preventing live activity products poisoning.

## Background

Microorganisms are detected in various conditions in most places [1]. They are the most ancient inhabitants of the planet. Due to their high variability, they have adapted to almost all extreme environmental niches, including high-temperature conditions [2]. They exist mainly in extreme environments. Microbial mats, in most cases autotrophic, are usually benthic communities typically formed on solid substrates that use CO_2_ as the carbon source. They are considered analogues of fossil stromatolites found in geological strata formed 3.5 billion years ago [3].

Microbial mats are good model systems for ecological and evolutionary analysis of microbial communities. They are usually small and almost closed self-sustaining ecosystems that include cycles of basic chemical elements and food chains. Sharp and continually changing gradients of physicochemical and chemical conditions create a large number of ecological micro-niches with very heterogeneous environments. The typical layered structure of phototrophic microbial mats is formed under the influence of the gradient of sunlight energy and chemical conditions supported by the activity of microorganisms [4, 5]. The most important function of phototrophic microorganisms in microbial mats is to absorb sunlight energy and CO_2_ to create organic material, including extracellular polymers [6]. Polymers forming extracellular matrices are very important for maintenance of microbial communities. They stabilize the sediments and the physical mat structure [7]. Organic material formed by primary producers is the basis for community food chains. It is converted by heterotrophic microorganisms of the community in various processes to produce energy and biomaterials [8].

There are several alkaline hot springs on the banks of the Barguzin river inflows. Water temperature reaches 75 °C and pH 8.0-9.0. The formation of microbial mats is observed in all hot springs [9]. Microbial communities of hot springs of the Baikal rift zone are poorly studied, especially using modern methods of molecular biology. In most cases, methods of microbiology were used to describe them. Only the communities of microbial mats of the Alla hot springs were described using molecular biology methods [10].

In this work, we studied а microbial mat detected in the Garga hot spring of the Baikal rift zone of the bank of the Garga River. The Garga hot spring differs from other hot springs of the Baikal rift zone, which have lower pH values (8.1). The spring comes to the surface as a single outlet, not a group of small sources like most hot springs of the Barguzin river valley. The microbial mat is formed on the slopes of the travertine dome. The thickness of the structured microbial mat is up to 7 cm.

## Methods

### Sampling

Samples were taken from the Garga hot spring (Barguzin valley, East Barguzin fault, Baikal rift zone, Russia) on June 5–8, 2010. Samples of microbial communities for metagenomic studies were collected in sterile 50-ml Falcons and fixed with 96% ethanol. The samples were stored at −72 °C in "Collection of biotechnological microorganisms as a source of novel promising objects for biotechnology and bioengineering of Federal Research Center Institute of Cytology and Genetics of the Siberian Branch of the RAS".

The spring is located on the slope on the western side of the Barguzin valley. It forms a carbonate “travertine” dome consisting of several terraces with the maximum thickness of 2.5 m; terraces are 30 to 80 cm high [11, 12]. A thick cyanobacterial mat covers the travertine surface of the spring bank.

The sulphate-sodium water of the Garga hot spring has mineralization of 1 g/l. SiO_2_ content is 30 mg/l; F, 11 mg/l; the highest temperature, 77 °C; and pH, 8.1. Microelement composition is dominated by Li, Rb, Sr, Ca, and Ba with lower amounts of Ge, Mo, and W [13]. Geology and hydrology of the spring were described in detail in [11, 14–16].

### DNA extraction

DNA extraction was performed in September 2010. The sample was triturated in a sterile ceramic mortar if necessary. Approximately 300 μl of sample suspension was added to Eppendorf tubes (2 mL). The suspension was centrifuged at 8000×g for 10 min. The pellet was re-suspended by pipetting in 500 μl buffer containing 100mMol Tris-HCl, pH 8, 100mMol EDTA, pH -8.0; 30 μl of chloroform and 200 μl of lysozyme (50 mg/ml) were added, and cells were incubated at 37 °C for 1 h, with shaking at 5 min intervals, and with subsequent addition of 100 μl 10% SDS and 100 μl 10% sarkosyl (Sigma). We performed three cycles of freezing in liquid nitrogen for 2 min and thawing for 5 min at 65°С. Another freeze-thaw cycle was performed after addition of 100 μl 10% Polyvinylpyrrolidone (Sigma). Then 50 μl of 2.5М СаСl_2_ were added, and the suspension was incubated at 65°С for 10 min with occasional shaking. The supernatant was transferred to a sterile tube, and an equal volume of phenol/chloroform (1/1) was added to the tube. The tubes were vortexed for 2 min and centrifuged at 13,000×g for 10 min. The supernatant (400 μl) was transferred to a new tube containing 100 μl 10 M NH_4_Ac and 1 ml of 96% ethanol, incubated overnight at −20 °C and centrifuged at 16,000×g for 20 min. The precipitate was washed with 70% ethanol, dried at room temperature and dissolved in water (mQ).

### Library construction

DNA extracted from the samples was used as a template for amplification of bacterial and archaeal 16S rRNA genes with universal primers: U341F (5’-CCTACGGGRSGCAGCAG-3′) and U806R (5′- GGACTACNVGGGTWTCTAAT-3′) [17, 18]. Reagents for PCR (DMSO, PCR buffer, polymerase, nucleotide triphosphates) were purchased from Agilent Technologies, USA. PCR mix (50 μl) contained 1× herculase buffer, 10 μM of each dNTPs, 10pmoles of forward and reverse primers, 100 ng of DNA, and 2.5u of Herculase. The following amplification profile was used: 3 min at 95 °C; 6 cycles of 15 s at 95 °C, 15 s at 50 °C, and 60 s at 72 °C; 35 cycles of 10 s at 95 °C, 10 s at 55 °C, and 60 s at 72 °C; and an additional elongation phase of 5 min at 72 °C. Amplified products were purified using commercial kits (Fermentas, Lithuania) and used for an additional PCR reaction with primers containing marker sequences designed for the 2x250bp read lengths sequencing protocol, according to the manufacturer’s instructions (Illumina, USA). The following re-amplification profile was used: 3 min at 95 °C; 4 cycles of 15 s at 95 °C, 15 s at 56 °C, and 60 s at 72 °C; 15 cycles of 10 s at 95 °C, 10 s at 55 °C, and 30 s at 72 °C; and an additional elongation phase of 5 min at 72 °C. The obtained PCR fragments for the different samples were mixed and purified by electrophoresis in 1,5% agarose gel. Libraries were constructed in October 2012.

### NGS sequencing

NGS sequencing of the variable V3-V4 regions of the 16S rRNA gene was performed on MiSeq (Illumina) using the MiSeq reagent kit v.2 (Illumina). Library preparation was done with Nextera DNA sample prep (Illumina). Sequencing was performed at the Faculty of Bioengineering and Bioinformatics, Lomonosov Moscow State University. Sequencing was performed in April 2013.

### Metagenomic data processing

16S rRNA reads (a total of 232,310 reads) were filtered, denoised and processed with a QIIME pipeline v1.9.1 [19]. All sequences were clustered, de novo chimera checked and quality filtered using USEARCH (Ultra-fast sequence analysis) tool v5.2.236 [20] against the Gold database (http://drive5.com/uchime/gold.fa). Sequences tagged as non-chimeric were combined and sorted by abundance. Then, operational taxonomic units (OTU) picking was performed; each non-chimeric read was assigned to a specific OTU identifier. A representative sequence for each OTU was queried against the GREENGENES database v13_8 [21] using UCLUST v1.2.22q program (http://www.drive5.com/uclust/downloads1_2_22q.html) from QIIME. The represented OTU was submitted in NCBI (KY767848-KY767913, KY552127-KY552260, KY552040-KY552126, KY551710-KY551870, KY551585-KY551709, KY551871-KY552039, KY497789-KY497909, KX979916-KX980027).

### Phylogenetic tree construction

Reference sequences of microorganisms’ 16S rRNA genes were obtained from the NCBI database (refseq_rna). Phylogenetic analysis was performed using MEGA (molecular evolutionary genetics analysis; http://www.megasoftware.net/index.html.), version 6.0 [22]. Distance matrices were calculated according to the Kimura two-parameter model [23]. Phylogenetic trees were inferred using the neighbour-joining method [24]. Bootstrap values were determined based on 1000 replications.

## Results and discussion

**Fig. 1:**
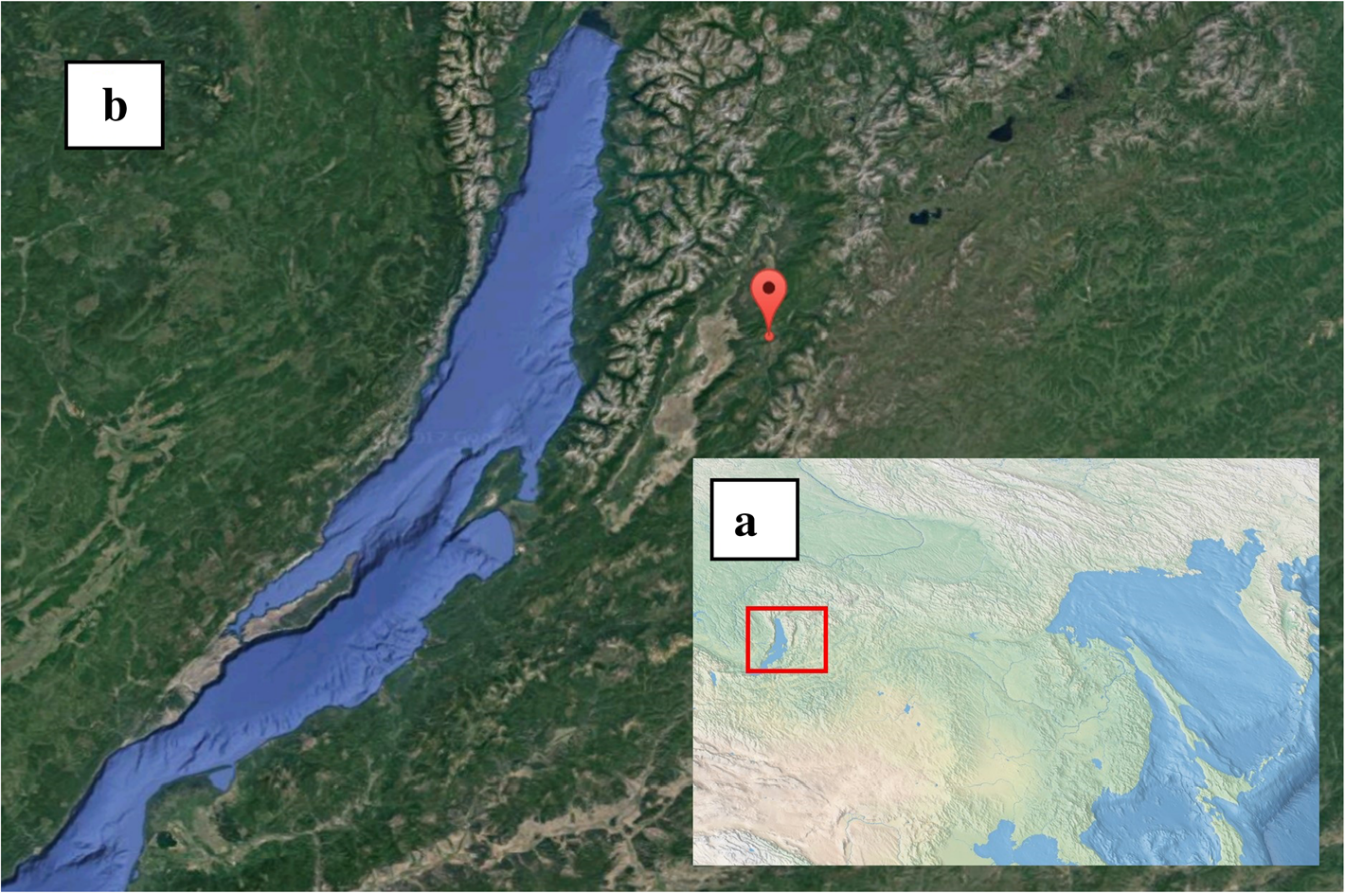
**a**, Eastern Siberia; **b**, Eastern Baikal, Barguzin Valley, satellite photo. Red cross marks the location of the Garga hot spring (54°19′3.72″N, 110°59′38.4″E)

**Fig. 2:**
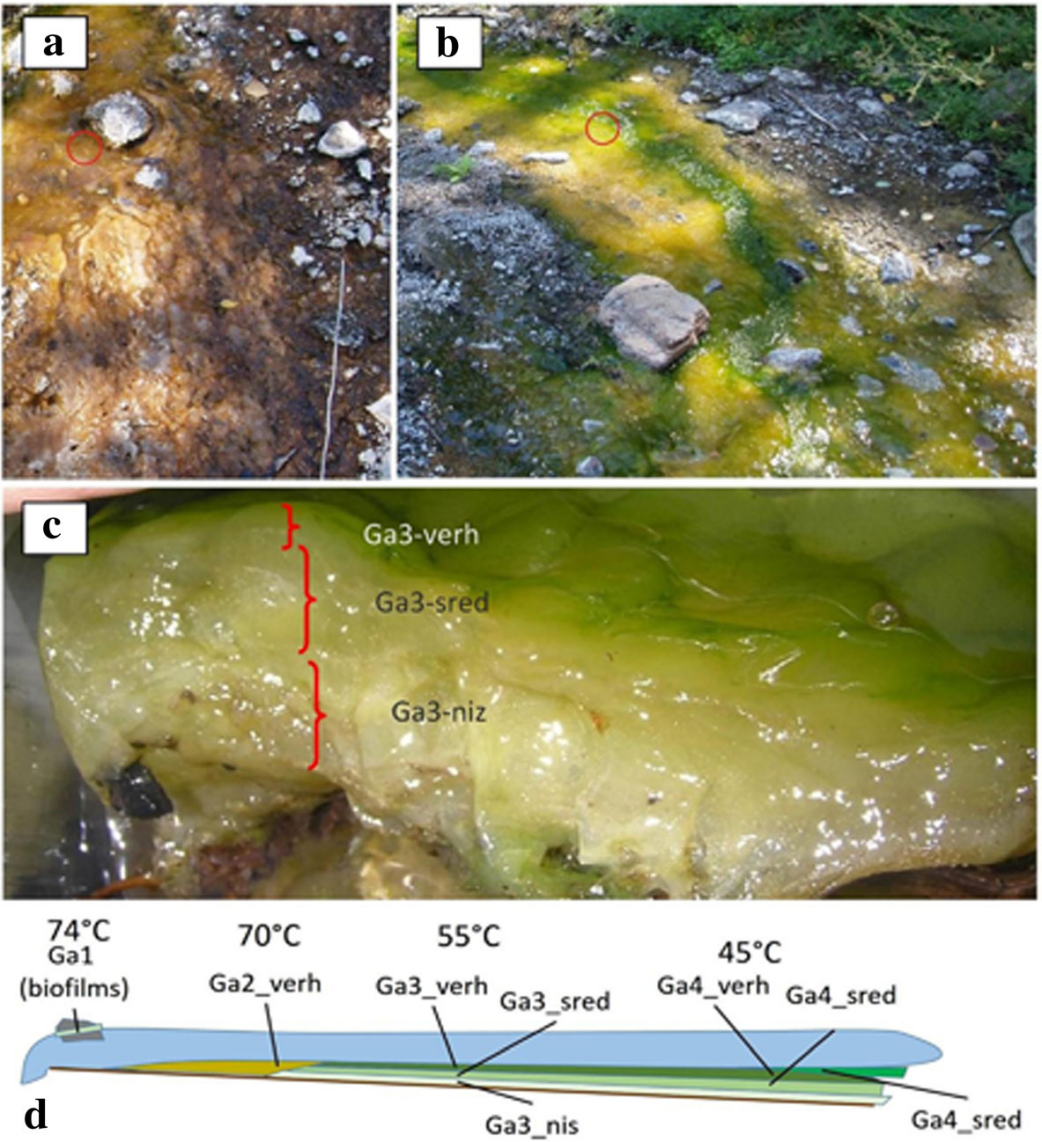
Microbial mats of the Garga hot spring. **a**, microbial mat of the upper reaches of the spring; red circle, GA2 sampling point. **b**, microbial mat of the middle reaches; red circle, GA3 sampling point. **c**, layers of the GA3 sample. **d**, sampling scheme

The Garga hot spring (Fig. 1) has pH 8.1, unlike most hot springs of this region that have pH closer to 9.0. Microbial mats (Fig. 2) in the Garga hot spring formed in the stream from the exit to the surface to the confluence with the Garga river (the inflow of the Barguzin river).

### The description of the Garga hot spring microbial mats and the selected samples

Sampling scheme is shown in Fig. 2d.

GA1 point was located in the area of the thermal water exit. The temperature at the time of sampling was 74 °C. A small white biofilms placed on the rocks (sample Ga1).

GA2 point (Fig. 2a) was located in the thermal stream with the temperature about 70 °C. At the edge of the stream, we observed a yellow gelatinous microbial mat. This mat extended from this point to the point of confluence into the river. The mat was thick (4 сm), very tight and layered. The top layer of the mat (sample Ga2_verh) was a thin (2 mm) film strongly linked to the underlying layer. The middle layer was less dense. The thickness of that layer was 2 cm. The bottom layer was white, gelatinous and linked to the substrate. The layer thickness was 1 cm.

Microbial mat in the GA3 sampling point ~55 °C (Fig. 2b, c) was dense, greenish, and 2-3 cm thick. The top layer (sample Ga3_verh) was a yellow-green film approximately 2 mm thick, hardly separable from the middle layer. The middle layer (sample Ga3_sred) was membranous, gelatinous and whitish-green. The upper two layers were similar in structure to the microbial mat of point GA2, but had a more intensive green colour. The bottom layer of the mat (sample Ga3_niz) was white, attached to the substrate, with a thickness of about 1 cm. The profile picture of the microbial mat at that point is shown in Fig. 2c.

The mat in the sampling point GA4 45 °C. The upper two layers (samples Ga4_verh and Ga4_sred) were similar to the microbial mat of point GA3. The bottom layer of the mat at that point was a relatively thick “film”—skin coloured, with a thickness of 0.3-0.5 cm. In addition to the structured microbial mats at this point, we also revealed friable bright green films (sample Ga4_zel).

**Fig. 3:**
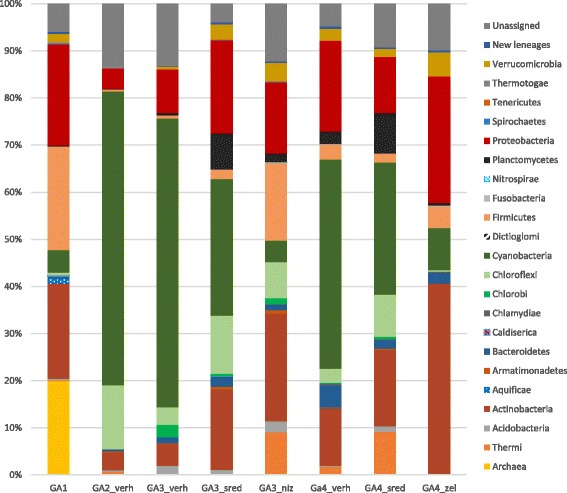
The percentage of various microorganism groups in the samples

After bioinformatics processing of metagenomic data, we obtained from 13,000 to 34,000 16S rRNA sequences for each of the eight studied samples. The total number of sequences was 222,201. Sequences were divided into operational taxonomic units (OTUs) individually for each sample; 5% to 15% of sequences in each sample could not be identified. Figure 3 shows the number of various microorganism types. Data were submitted in NCBI (KY767848-KY767913, KY552127-KY552260, KY552040-KY552126, KY551710-KY551870, KY551585-KY551709, KY551871-KY552039, KY497789-KY497909, and KX979916-KX980027). FASTQ files are in supplement.

#### Microbial communities of the sampling point with highest temperatures (GA1)

In the hottest point of the Garga hot spring (GA1) 74 °C, biofilms were observed on the border with the air. Figure 3 shows the relative abundance of various microorganisms in this point (Ga1). Phototrophic microorganisms accounted for only about ~6% of the total sample, while Firmicutes (Bacilli) (21.9%) and Proteobacteria (21.4%) were the most abundant, the former represented mostly by the mesophilic *Bacillus pumilus* [25]. Approximately 3% of sequences belonged to the genus *Staphylococcus*.

#### Archaea

**Fig. 4:**
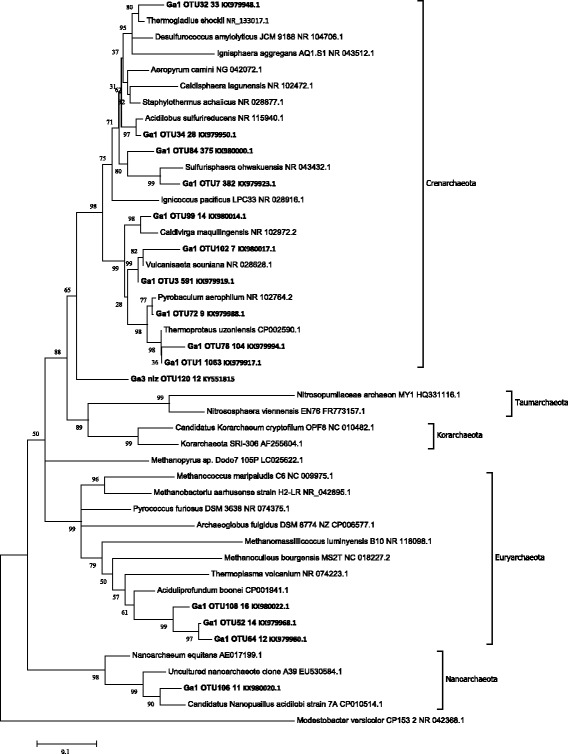
Maximum likelihood tree of Archaea obtained in this study. Bootstrap values were calculated for 200 alternative trees; scale, number of substitutions per site. OTU names include sample name, OTU number, and the number of sequences belonging to this group

Archaea constituted 19.8% of the total number of sequences, which is the highest percentage for the Baikal rift zone obtained to date. Archaea were not detected in other sampling points except Ga3-sred (0.04%). Only OTU-1, OTU-34 and OTU-72 belonging to Crenarchaeota were closely related to any cultivated archaea (Fig. 4). The remaining archaeal sequences obtained in our study had no close similarity to any known microorganisms, as well as in most cases to sequences of uncultivated specimens.

Crenarchaeota was the most abundant archaeal phylum in our sample. OTU-1 (7.9% of the total archaeal sample) was very similar (99%) to *Thermoproteus uzonensis* [26]. This anaerobic archaeon was isolated from the Uzon volcano caldera in the Kamchatka peninsula. Its optimum growth was observed at pH 5.5, while the Garga hot spring has pH 8.3. OTU-3 (4.4%) had 99% sequence similarity to the crenarchaeote *Vulcanisaeta souniana* isolated from several hot spring areas in eastern Japan, which is also anaerobic and acidophilic [27]. OTU-7 and OTU-84 (2.8% each) had no close sequence similarity to any known species. Metagenomic sequences with the highest degree of similarity (96%) for OTU-7 were found in hot springs in Japan [28] and Thailand (NCBI/nr), while for OTU-84, 97% similar sequences were obtained from hot springs in Iceland [29]. OTU-78 (0.7%) had only 92% sequence similarity to uncultured microorganisms. Another 9 archaean OTUs were less abundant and had less than 95% sequence similarity to known microorganisms, which means that hot springs of the Baikal region contain many new species of Archaea.

### Microbial communities in the microbial mats of Garga hot spring

#### Cyanobacteria

**Fig. 5:**
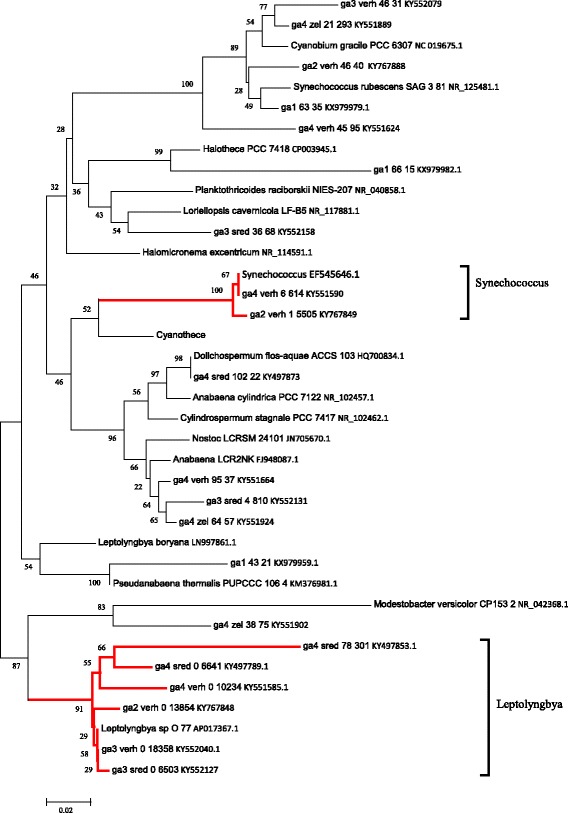
Maximum likelihood phylogenetic tree of cyanobacterial OTUs found in the Garga hot spring. Bootstrap values were calculated for 200 alternative trees; scale, number of substitutions per site. OTU names include sample name, OTU number, and the number of sequences belonging to this group. Branches including highly abundant OTUs are shown in red

Cyanobacteria (Fig. 5) dominated in the microbial mat of the Garga hot spring community (points GA2, GA3, and GA4). They produce much of the organic matter and form the structure of the microbial mat. *Leptolyngbya* accounted for the majority of cyanobacterial sequences (> 70%) in all microbial mat samples. All sequences of *Leptolyngbya* were most closely related to that of *Leptolyngbya* sp. O-77 isolated from Japanese hot springs [30]. Representatives of the genus *Leptolyngbya* were detected in hot springs in Romania [31], Teng Chong (China) [32], Neuquen (Argentina) [33], and Yellowstone (USA) [34]. They were prevalent in microbial mats growing in alkaline fluids in northeast Australia [35], East Africa [36], and Greenland [37], as well as in certain hot springs in the Baikal region [10].

Representatives of the genera *Synechococcus* (OTU-1) were also abundant in Ga2-verh, and *Nostoc* (OTU-4), in Ga3-sred. OTU-1 (17.5%) had 99% sequence similarity to the organisms found in the Alla and Uro hot springs (NCBI/nr) near Baikal. OTU-4 (9.0%) was 96% similar to a cyanobacterium isolated from a microbial mat in a cement factory in India (NCBI/nr KF746950, KF746951). The genera *Synechococcus* and *Nostoc* are widespread in microbial mats of most hot springs. *Synechococcus* is a cosmopolitan genus of cyanobacteria found in marine, freshwater, thermal, terrestrial and subaerial habitats [38, 39]. This genus is the most polyphyletic group of cyanobacteria and in the future a possible splitting of the Synechococcus lineages into different genera [40].

Algal (Stramenopiles) 16S rRNA sequence were also detected in the microbial mat. Algae probably provided bright green colour to the mat at point GA4. Stramenopiles were found in various marine symbiotic communities, e.g., inside the sponge *Tethya californiana* [41]. The photosynthetic stramenopile Ochrophyta forms a highly diverse clade within Heterokonta, a clade that also included a number of heterotrophic lineages such as plant moulds and aquatic pseudofungi. The majority of published molecular phylogenetic analyses indicate that the photosynthetic and non-photosynthetic stramenopiles form a monophyletic taxon [42]. The earliest fossil remains (Palaeovaucheria; Xanthophyceae) suggest that the photosynthetic stramenopiles appeared 1000 million years ago (Ma) [43].

#### Chloroflexi and Chlorobi of the Garga hot spring microbial mats

**Fig. 6:**
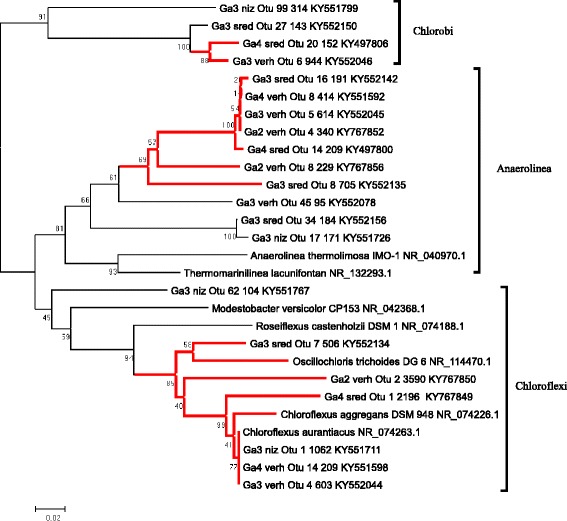
Maximum likelihood phylogenic tree for OTU representative sequences of anoxygenic phototrophs found in the Garga hot spring. Bootstrap values were calculated for 200 alternative trees; scale, number of substitutions per site. OTU names include sample name, OTU number, and the number of sequences belonging to this group. Branches including highly abundant OTUs are shown in red

Although layers formed by Chloroflexi and Chlorobi were not detected, they were identified in all our samples. They accounted for > 10% of the total number of sequences in the Ga2-verh and Ga3-sred samples and were most closely related to the organisms from the same rift zone, the Alla and Uro hot springs [10]. Chloroflexi are facultative anaerobes, anoxigenic phototrophs or lithotrophs [44, 45]. Chlorobi are anaerobic and anoxic phototrophs that can use sulfide and thiosulfate as electron acceptors [46]. We found two groups of Chloroflexi formed by the genera *Chloroflexus* and *Anaerolineae*, as well as one group comprised of various Chlorobi (Fig. 6). Representatives of the Chloroflexus group were closely related to the well-known *Chloroflexus aurantiacus* [45, 47]. The rest of the algae had less than 83% sequence similarity to known microorganisms (NCBI/refseq_rna). Chloroflexi and Chlorobi are widespread in microbial mats in hot springs in Japan [48], Yellowstone (USA) [49], Kamchatka [50], Thailand [51], Tibet [52], and Andes [53].

#### Heterotrophic microorganisms of the Garga hot spring microbial mat

Proteobacteria and Actinobacteria prevailed among heterotrophic microorganisms in all samples of the Garga hot spring microbial mat. Actinobacteria were mainly represented by two orders, Acidimicrobiales and Actinomycetales. Diversity of Proteobacteria was higher and was mainly represented by the orders Rhizobiales, Rhodobacterales, Rhodospirillales, Sphingomonadales, Burkholderiales, Pseudomonadales, and Xanthomonadales.

Proteobacteria were found in almost all studied hot springs, including Andes, Colombia [54], South Africa [55], Kamchatka (Mutnovsky, caldera Uzon) [56], Malaysia [57], Tengchong (China) [32], Romania [31], Spain [58], and Yellowstone (USA) [59].

Actinobacteria were found in microbial communities in hot springs of Kamchatka (Russia), Tengchong (China), Nevada (USA) [60], Bor-Khlueng (Thailand) [61], Japan [62], and many others; however, they never prevailed.

Actinobacteria are the most numerous organisms of soil and aquatic ecosystems [63, 64]. They play an important role in geochemical cycles. They are gut symbionts [65] and animal pathogens [66]. Actinobacteria are interesting for biotechnology as destructors of plant residues [18] and producers of a large number of secondary metabolites [67]. *Thermobifida fusca* and *Acidothermus cellulolyticus* 11B [68] are the most famous among thermophilic Actinobacteria due to the presence of cellulolytic enzyme complex. Nevertheless, information on the biodiversity and role of Actinobacteria in geothermal habitats is scarce [69, 70].

The middle and bottom layers of the Garga hot spring microbial mat consist of significant amounts of other types of heterotrophs except for Proteobacteria and Actinobacteria. In the middle layer of the microbial mat in the Ga-3-sred and Ga-2-sred samples, Planctomycetes accounted for 7% to 9% of the total number of sequences. Verrucomicrobia formed up to 5% in GA3 (except Ga-3-verh) and GA4. Planctomycetes and Verrucomicrobia were reported for many geothermal springs [31, 57, 71].

Firmicutes (mainly *Clostridia*) accounted for 14.6% of the total number of sequences in the Ga-3-niz layer of the GA3 point (Fig. 3). *Clostridia* are strong anaerobes. The sequences of this class were not found in other samples. The thermophilic *Clostridia* are best known for their ability to degrade lignocellulose. They form cellulosomes, enzymes united in a macromolecule via interaction of special domains (Cohesines and Dockerines) and providing strong binding of subunits [72].

Thermi is another type of bacteria that was abundant in the microbial mat (samples Ga-3-niz and Ga-4-sred). Bacteria of this type are known as thermophiles and are distributed in hot springs everywhere [73].

#### Metabolism of Garga hot spring microbial mat

**Fig. 7:**
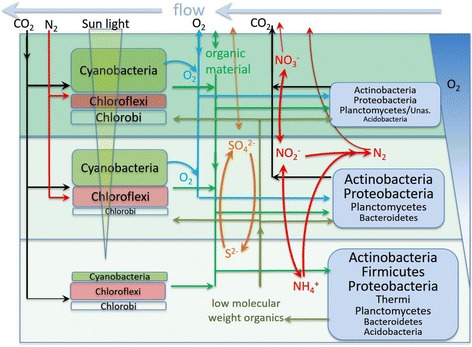
A scheme of the Garga hot spring microbial mat, including main groups of microorganisms and the pathways of substance and energy flow. The most abundant organism types are highlighted by bold and large print: green, cyanobacteria; pink, Chloroflexi; blue, heterotrophic bacteria

Metabolic pathways in the Garga hot spring microbial mat are based on the interaction of autotrophic and heterotrophic microorganisms and on the redox gradient. The analysed microbial mat can be divided into three layers (Fig. 7). Photosynthetic activity is the highest in the upper layer, while in the middle layer, the amount of sunlight decreases, as does the exchange rate of low molecular weight substances with water flow. As the rate of photosynthesis decreases, diversity and abundance of heterotrophic microorganisms increases. We observed an increase in the proportion of Chloroflexi in this layer, which may be associated with low sunlight intensity and the presence of redox gradient. No separate layer of anoxic phototrophic bacteria was observed, which may be associated with the high speed of surface water flow and smooth gradient redox.

The bottom layer of the microbial mat is the destruction zone. The oxygen does not penetrate here and is not produced by photosynthesis as evidenced by the presence of obligately anaerobic Clostridia in the bottom layer (Ga-3-niz). Phototrophic microorganisms and aerobic bacteria badly adapted for anaerobic conditions die in this layer. The organic material is destroyed to low molecular weight organics.

Anaerobic fermentation is the main mechanism for conversion of organic substances in the bottom layer of the microbial mat. Low molecular weight products formed by fermentation are moved into the upper layers, where they are aerobically oxidized and provide extra food for microorganisms living there. The concentration of inorganic substances in the water of the spring is too low, primarily of reduced sulphur compounds, so the probability that lithotrophic transformation pathways dominate in the Garga hot spring microbial mat is relatively low [13]. However, it should be noted that sulphur may be accumulated in different forms depending on the redox state, so a sulphur cycle may exist in the microbial mat. The catalysis of the sulphur redox conversion may be carried out by Proteobacteria, Firmicutes [33], Chloroflexi, and Chlorobi [74, 75].

According to geochemical analysis, the water of the spring does not contain nitrogen compounds, so nitrogen fixation is the only possible source of nitrogen for the community. We found many species capable of nitrogen fixation (both oxygenic and anoxygenic phototrophes, some representatives of Proteobacteria and Actinobacteria). The presence of Planctomycetes indicates active redox transformations of nitrogen compounds in the mat. They can oxidize ammonium produced in the bottom layer of the microbial mat, while subsequent reactions result in formation of N2 and removal of nitrogen from the cycle [76].

## Conclusion

High abundance of Archaea in samples from hot springs of the Baikal rift zone supplemented our knowledge of the distribution of Archaea. Most archaeal sequences had low similarity to known Archaea. We detected archaeal sequences that accounted for 19.8% of the total number of sequences in the Ga1 sample. It is first time when such amounts of Archaea were detected in samples from hot springs of the Baikal rift zone. We were the first to demonstrate abundance of Archaea in the hot springs of the Baikal rift zone. They could be delivered at the Ga1 point from the depth by the flow, while mesophilic bacteria could be from the surrounding microbial communities. The most abundant Archaea belonged to *Thermoproteus uzonensis* and *Vulcanisaeta souniana*. Those Archaea are anaerobic and acidophilic. Most detected archaeal OTUs did not have high similarity to known archaeal species.

Metagenomic analysis of microbial communities of the microbial mat of Garga hot spring showed that the three studied points sampled at 70 °C, 55 °C, and 45 °C had similar species composition. Cyanobacteria of the genus *Leptolyngbya* dominated in the upper layer of the microbial mat, accounting for over 60% of sequences, and considering that Cyanobacteria have large cells, their biomass share can exceed 90%. Chloroflexi and Chlorobi were less abundant and were mostly observed in the middle part of the microbial mat. Based on metabolic analysis, we suggest that there are complete cycles of carbon, sulphur, and nitrogen in the community. The carbon cycle starts with the formation of phototrophic biomass in the top layer with subsequent decomposition in the bottom layers to alcohols and organic acids, which are used by microorganisms in upper layers. Cycles of sulphur and nitrogen are complete due to the presence of redox gradient. The sulphur cycle can be considered a closed one; the outflow of sulphur compounds is carried out with the flow of water. Nitrogen compounds are removed from the cycle by diffusion and the anamnox reaction (NH_4_^+^+NO_2_^−^ = N_2_ + 2H_2_O) performed by Planctomycetes. Microbial mats evolved in early stages of biosphere formation. They can be considered a model of the systems that existed before the origin of plants. Our study demonstrates that microbial mats that evolved in early stages of biosphere formation could live autonomously, providing full cycles of elements and preventing poisoning by their own by-products.

Current thermal microbial mats are isolated from each other by mesopilic environments, and so may provide important insights into microbial evolution. Eukaryotes do not have significant effects on such communities because they are not adapted to high temperatures.

In addition to the Garga hot spring, there are other hot springs in the Baikal region, the microbial communities of which were poorly studied by modern genetic methods. This is one of the first studies on the detailed composition of a microbial mat from the Baikal area.
